# The Driving Forces of Point Source Wastewater Emission: Case Study of COD and NH_4_-N Discharges in Mainland China

**DOI:** 10.3390/ijerph16142556

**Published:** 2019-07-17

**Authors:** Zhaofang Zhang, Weijun He, Juqin Shen, Min An, Xin Gao, Dagmawi Mulugeta Degefu, Liang Yuan, Yang Kong, Chengcai Zhang, Jin Huang

**Affiliations:** 1 Business School, Hohai University, Nanjing 211100, China; 2 College of Economic & Management, Three Gorges University, Yichang 443002, China; 3 Faculty of Engineering and Architectural Science, Ryerson University, Toronto, ON M5B 2K3, Canada

**Keywords:** point sources, wastewater pollutant emissions, LMDI, spatial and temporal disparities, decomposition analysis

## Abstract

Excess consumption of water resources and environmental pollution have become major challenges restricting sustainable development in China. In order to prevent the pollution of water resources, policymakers should have reliable emission reduction strategies. This paper aims to contribute new knowledge by analyzing the spatial-temporal characteristics and driving forces of point source emission. The chemical oxygen demand (COD) and ammonia nitrogen (NH_4_-N) emission variations in 31 provinces and municipalities of mainland China during the years 2004–2017 are analyzed. The results obtained using the logarithmic mean Divisia index (LMDI) method indicate that: (1) the COD and NH_4_-N emission effects have similar temporal characteristics. Technology improvement and pollutant emission intensity are the main factors inhibiting the incremental COD and NH_4_-N emission effects, while economic development is the main driving factor of COD and NH_4_-N emission effects. Population increases play a relatively less important role in COD and NH_4_-N emission effects. (2) The spatial features of COD and NH_4_-N emission effects show differences among provinces and municipalities. The reduction of COD emission effects in each province and municipality is obviously better than that of NH_4_-N emissions. (3) In the eastern, central, and the western regions of China, the total COD emission effect shows a downward trend, while apart from the central region, the NH_4_-N emission effect appears to be rising in the east and west of China. Therefore, increasing investment into pollution treatment, promoting awareness of water conservation, strengthening technological and financial support from the more developed eastern to the less developed central and western regions, can help to reduce the COD and NH_4_-N emissions in China.

## 1. Introduction

China is the largest developing and fastest growing country [1,2]. The population, industrialization and urbanization rate have increased rapidly and caused large wastewater emissions which have become a major environmental concern [3,4]. According to the 2018 China Water Resources Bulletin, the I~III quality level of rivers comprise 78.5% of the total 245,000 km of the river network in China, whereas rivers worse than the V level account for 8.3%. In addition, the I~III class lakes cover only 26.0%, while IV~V lakes account for 74% (Ministry of Water Resources of the People’s Republic of China) [5]. Chemical oxygen demand (COD) and ammonia nitrogen (NH_4_-N) have become the main pollutants [6,7,8], along with the continuous increase of other emerging pollutants which will inevitably cause environmental and human health damages [9,10,11]. Heavy water pollution would hinder the construction of China’s ecological civilization and undermine the foundations for implementation of sustainable development [12,13], also it would aggravated the water shortage [14,15]. Therefore, finding ways to improve water use efficiency, reduce emissions and treat wastewater has become the main attention of the government [16].

Water pollution can be divided into point source pollution and non-point source pollution [17]. Point source pollution refers to emissions from pollution sources with a fixed discharge point, mainly from industrial wastewater pipes and urban sewage pipes. Non-point source pollution refers to the pollution without a fixed discharge point, usually referring to pollution from agriculture. Compared with non-point source pollution, point source pollution is characterized by high pollutant concentrations, large influence and various types of pollutants [18]. Meanwhile, the emission reduction of industrial and domestic sewage involved in point source pollutants is closely related to economic development. Therefore, it is important to study the driving factors of wastewater emission so that policymakers can formulate effective emission reduction strategies to control water pollutant emissions [19,20].

There are numerous studies on the factors that influence wastewater emissions. Some researchers use the environmental Kuznets curve (EKC) to verify whether the relationship between wastewater emission levels and economic growth is an inverted U-shape and to predict their turning point [21,22,23]. Other researchers use vector auto-regression (VAR) to study the association between wastewater discharge and economic growth [24,25]. The input–output model is used by Tang [26] to determine the key production chain of industrial wastewater discharge. However, these studies only focused on economic factors that affected wastewater emissions, and ignored social factors that might be equally important. Decomposition analysis is a good way to identify key driving factors of wastewater discharge [27]. Compared with the previous decomposition methods, the logarithmic mean Divisia index (LMDI) [28,29,30] replaces the arithmetic average weight with the logarithmic average weight, which could eliminate the residual error after decomposition making the decomposition result more favorable. Moreover, LMDI can process the zero value in data which doesn’t affect the final result [31,32,33]. Hence, the LMDI method has been applied to identify key driving factors of wastewater discharge in China at the provincial level [19], industrial wastewater emissions [34,35], household wastewater emissions [36] and the COD discharge in China’s industrial sub-sectors [37,38]. These studies explore the driving factors of China’s wastewater emission change under different time scales, including economic scale, industrial structure, technological progress, and population size, and proposing industry or regional emission reduction strategies. However, most research focuses on a single industry’s wastewater or a single pollutant, without an in-depth and comprehensive consideration of the influencing factor of multi-industry wastewater discharge pollutants. There is a lack of a more in-depth research on point source water pollution.

At the same time, China is a country with a large gap in regional socio-economic development [39]. The studies above tend to focus on one province or one municipality, thus ignoring regional heterogeneity in terms of wastewater discharge. At the provincial scale, there is still a lack of studies focusing on the emissions of specific wastewater pollutants’ driving factors. Hence, further studies are needed so as to comprehensively and incisively figure out the spatial-temporal characteristics of COD discharge and NH_4_-N emission. In this article, to fill in these research gaps, based on the LMDI method, the influencing factors of COD discharge and NH_4_-N emission changes in various provinces in China are divided into the pollutant emission intensity, wastewater technology improvement, economic development, and population increases. Then, the spatial-temporal characteristics and driving factors underlying changes with 31 provinces and municipalities in mainland China are analyzed. The rest of this paper is arranged as follows. Section 2 describes the research method and presents data collection. Section 3 shows the main results and discussion. The conclusion and recommendations are offered in Section 4.

## 2. Methods

### 2.1. Logarithmic Mean Divisia Index (LMDI) Model

Point source wastewater emissions are mainly influenced by the intensity of pollutant emissions, technological progress, economic activities and population growth [34,38]. This study explores the influential factors of the point source wastewater emissions by the LMDI method. According to Kaya [40,41,42], total wastewater discharge can be expressed by the following Equation (1):

(1) $$W^{t}=\sum_{i}^{n}W_{i}^{t}=\sum_{i}^{n}\left[\left(\frac{W_{i}^{t}}{V_{i}^{t}}\right)\cdot \left(\frac{V_{i}^{t}}{G_{i}^{t}}\right)\cdot \left(\frac{G_{i}^{t}}{P_{i}^{t}}\right)\cdot P_{i}^{t}\right]$$

where $W^{t}$ represents the total amount of pollutant (COD or NH_4_-H) emitted in year t throughout the country; $V_{i}$ represents the water resource consumption in province i; $G_{i}$ represents the regional gross domestic product and $P_{i}^{}$ represents the total population in the province i. Where a positive value indicates that the index has a positive reinforcing effect on emission effects, and a negative value represents that the index has an inhibiting effect on emission effects.

To carry out this analysis, it is necessary to take into account the different factors affecting the causes that produce a change in wastewater pollutants. Equation (1) could be reduced to Equation (2).

(2) $$W^{t}=\sum_{i}^{n}\left(W_{S,i},W_{T,i},W_{E,i},W_{P,i}\right)$$

$W_{S,i}$ shows the pollutant emission intensity level which represents per unit of water resource that could bear the degree of pollution (COD or NH_4_-H), which represents the dilution effect of water. A lower value indicates the amount of water resource that will produce a better reduction effect on wastewater pollutants.

$W_{T,i}$ depicts wastewater technology improvement effect and is measured by the total consumption of every unit of water resource. The lower the ratio is, the more technology can help reduce total water pollution.

$W_{E,i}$ denotes the impact of economic development, which is designated by the regional gross domestic product and called economic development effect. The regional gross domestic product increase leads to high emission levels.

$W_{P,i}$ represents the population increase effect and indicates the influence of the population on wastewater discharge levels. If the ratio is higher, the larger population will produce more pollutant emissions.

According to the LMDI approach put forward by Ang [32,43], the change of wastewater pollutants between a base year m and a target year t, is denoted by $\Delta W^{t-m}$.

(3) $$\Delta W^{t-m}=\Delta W_{S,i}+\Delta W_{T,i}+\Delta W_{E,i}+\Delta W_{P,i}$$

The change in emission levels caused by each factor to wastewater pollutants can be calculated by the following formulas:

(4) $$\Delta W_{S,i}=\frac{W_{i}^{t}-W_{i}^{0}}{\ln W_{i}^{t}-\ln W_{i}^{0}}\cdot (\ln\frac{W_{S,i}^{t}}{W_{S,i}^{0}})$$

(5) $$\Delta W_{T,i}=\frac{W_{i}^{t}-W_{i}^{0}}{\ln W_{i}^{t}-\ln W_{i}^{0}}\cdot (\ln\frac{W_{T,i}^{t}}{W_{T,i}^{0}})$$

(6) $$\Delta W_{E,i}=\frac{W_{i}^{t}-W_{i}^{0}}{\ln W_{i}^{t}-\ln W_{i}^{0}}\cdot (\ln\frac{W_{E,i}^{t}}{W_{E,i}^{0}})$$

(7) $$\Delta W_{P,i}=\frac{W_{i}^{t}-W_{i}^{0}}{\ln W_{i}^{t}-\ln W_{i}^{0}}\cdot (\ln\frac{W_{P,i}^{t}}{W_{P.i}^{0}})$$

The above four formulas represent the influence of pollutant emission intensity, technology improvement, economy and population on the wastewater pollutants emission. Where a positive value represents that the index has an increasing effect on wastewater pollutant emission and a negative value represents that the index has an inhibiting effect on wastewater pollutant emission.

### 2.2. The Relative Contribution Rate of Each Factor

The relative contribution rate of each factor is the ratio of the relative value of each factor to the total impact of the COD or NH_4_-N emissions. A higher ratio indicates that the factor has a positive reinforcing role on pollutant’s emission effect, the factor will bring more effects on the increase of COD discharge or NH_4_-N emissions. Conversely, a smaller result means the factor has less of an influence on the final results. Where, $\omega_{S},\omega_{T},\omega_{J},\omega_{P}$ represents the relative contribution rate of each factor and $\Delta W_{R,i}$ reflects the relative total sum of each factor’s effect.

(8) $$\left|\frac{\left|\Delta W_{S,i}\right|}{\left|\Delta W_{R,i}\right|}+\frac{\left|\Delta W_{T,i}\right|}{\left|\Delta W_{R,i}\right|}+\frac{\left|\Delta W_{E,i}\right|}{\left|\Delta W_{R,i}\right|}+\frac{\left|\Delta W_{P,i}\right|}{\left|\Delta W_{R,i}\right|}\right|=\left|\omega_{S}+\omega_{T}+\omega_{E}+\omega_{P}\right|$$

(9) $$\Delta W_{R,i}=\Delta W_{S,i}+\Delta W_{T,i}+\Delta W_{E,i}+\Delta W_{P,i}$$

### 2.3. The Absolute Contribution Rate of Each Factor

The absolute contribution rate of each factor is the proportion of the absolute amount of the change of each factor to the sum of the absolute total effect, which represents the contribution degree of a factor. Specifically speaking, the larger the value is, the higher the contribution degree of this factor will be. Where, $\lambda_{S},\lambda_{T},\lambda_{J},\lambda_{P}$ represents the absolute contribution rate of each factor, and $\Delta W_{A,i}$ reflects the absolute total sum of each factor’s effect.

(10) $$\left|\frac{\left|\Delta W_{S,i}\right|}{\left|\Delta W_{A,i}\right|}+\frac{\left|\Delta W_{T,i}\right|}{\left|\Delta W_{A,i}\right|}+\frac{\left|\Delta W_{E,i}\right|}{\left|\Delta W_{A,i}\right|}+\frac{\left|\Delta W_{P,i}\right|}{\left|\Delta W_{A,i}\right|}\right|=\left|\lambda_{S}+\lambda_{T}+\lambda_{E}+\lambda_{P}\right|=1$$

(11) $$\Delta W_{A,i}=\left|\Delta W_{S,i}\right|+\left|\Delta W_{T,i}\right|+\left|\Delta W_{E,i}\right|+\left|\Delta W_{P,i}\right|$$

### 2.4. Study Area and Data Source

The data span used in this paper is from 2004 to 2017. The data on the water consumption, regional gross domestic product, regional populations and the total amount of water resources are collected from the China Statistical Yearbooks [44] and China Statistic Yearbook on Environment [45]. The COD and NH_4_-N emission data are obtained from the China Water Resources Bulletin [46]. Taking into account data availability, 31 administrative regions (27 provinces and four municipalities) of mainland China are considered in this study. The provinces and municipalities (under the direct administration of central government) are listed in Table 1.

## 3. Results and Discussion

### 3.1. Temporal and Spatial Trends of Water Resource Consumption and Pollution in Mainland China

#### 3.1.1. The Temporal Evolution of Water Resource Consumption and Pollution in Mainland China

**Figure 1:**
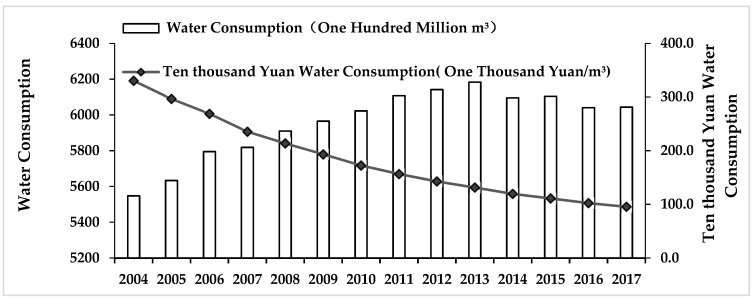
The total amount of water consumption and ten thousand Yuan water consumption of mainland China during 2004–2017.

Figure 1 shows the total water consumption and water intensity trends in mainland China from 2004 to 2017. In general, the total water consumption of mainland China variation presents increasing trends first and decreasing ones afterwards. It increased from 554.768 billion m^3^ in 2004 to 618.347 billion m^3^ in 2013, the total volume increased by 63.579 billion m^3^, with an average annual growth rate of 1.01%. However, from 2013 to 2017, the total water consumption displayed a downward trend with a total reduction of 14.07 billion m^3^, with an average annual reduction rate of 0.57%, which demonstrates China’s stringent effectiveness of its water resource management policy. The policy issued in 2013 has played a significant role in promoting water conservation.

Meanwhile, water intensity can be depicted by the water consumption of ten thousand Yuan which represents the water consumption per 10,000 Yuan of output value. According to Figure 1, the water consumption of 10,000 Yuan showed a decreasing trend from 2004 to 2017, from 330.4 in 2004 to 95.5 in 2017, with an average annual decline rate of 9.12%. This indicates that with economic development and technological progress, large water consumers such as industry have prompted increases in the water production value. 

**Figure 2:**
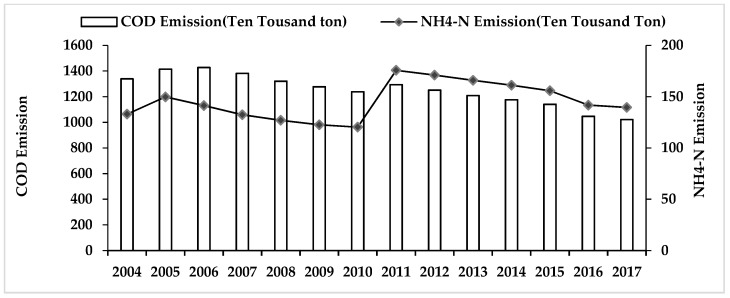
The total amount emissions of the COD and NH_4_-N in mainland China from 2004 to 2017.

As shown in Figure 2, the point source emission of the COD follows a fluctuating downward trend from 2004 to 2017, dropping from 13.3918 million tons in 2004 to 10.2198 million tons in 2017, with an average annual decline rate of 2.06%. Even though NH_4_-N emissions exhibited a phased decreasing trend during the time periods 2005–2010 and 2011–2017, overall emissions increased slightly, among which NH_4_-N increased from 1.3299 million tons in 2004 to 1.3951 million tons, with an average annual growth rate of 0.37%. At the same time, it is worth noting that an increasing trend in the emissions of COD and NH_4_-N was observed from 2004 to 2005 and 2010 to 2011. The possible explanation is the implementation of macroeconomic policies like the increase in investment, which had encouraged a large number of projects such as infrastructure construction and industrial projects [35,37]. For instance, in 2005, a serious water pollution incidents occurred in the Songhua river of Northeast China where about 100 tons of benzene were directly discharged, leading to a sharp increase in water pollution levels that year [47]. The increase in water pollution levels in 2011 was caused by China’s multiple investment programs, which stimulated the development of a new round of pollution-intensive industry and infrastructure [48].

#### 3.1.2. The Spatial Analysis of the Provincial Water Resource Consumption and Pollution in China

**Figure 3:**
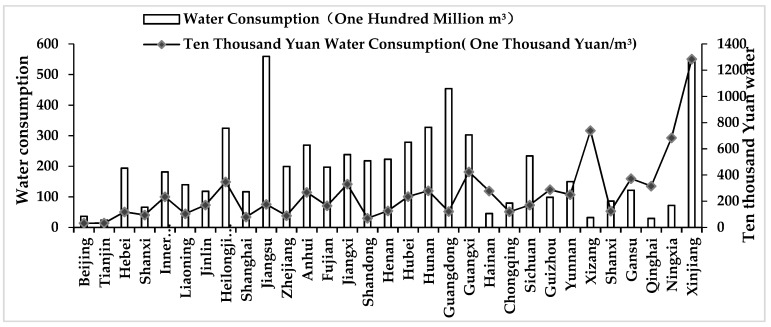
The average value of water consumption and water intensity in each province of mainland China from 2004 to 2017.

Figure 3 shows the average total water consumption and water consumption per ten thousand Yuan in provinces and municipalities for the time period of 2004 to 2017. The province with the greatest average water consumption is Jiangsu (55.924 billion m^3^), while the one with the smallest average water consumption is Tianjin (2.388 billion m^3^). Meanwhile, the standard deviation of total water consumption in the 31 provinces and municipalities of mainland China reaches 13.959 billion m^3^. The provinces with the largest and smallest water consumption of ten thousand Yuan are Xinjiang (68.365 billion m^3^) and Tianjin (3.279 billion m^3^), while the standard deviation of ten thousand Yuan water consumption in the 31 provinces and municipalities is 24.734 billion m^3^.

**Figure 4:**
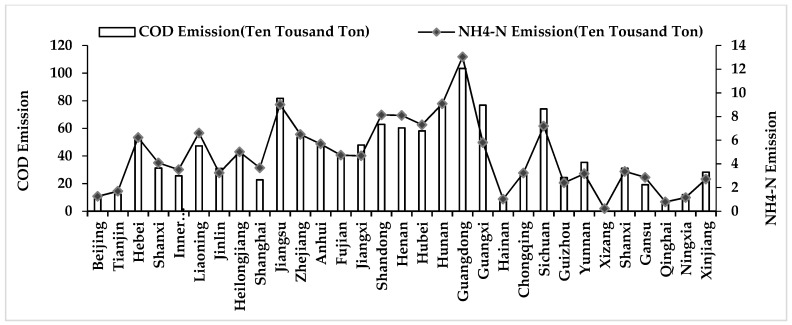
The average value of COD emissions and NH_4_-N emissions in each province of mainland China from 2004 to 2017.

According to Figure 4, the COD and NH_4_-N emissions in Guangdong proved to be the largest among all the provinces and municipalities, reaching pollution levels of 1,034,300 tons and 134,400 tons, respectively. In contrast, Xizang (Tibet) is the province with the lowest levels of emissions, with only 20,000 tons and 2200 tons, respectively. The standard deviation of COD emissions in the 31 provinces and municipalities was 247,900 tons, and that of NH_4_-N was 290,000 tons. It can be seen that there were significant spatial differences in the total water consumption levels and ten thousand Yuan of water consumption among the 31 provinces and municipalities in mainland China.

### 3.2. The Spatio-Temporal Analysis of Driving Factors of COD and NH_4_-N Emissions

#### 3.2.1. The Temporal Decomposition Analysis of COD and NH_4_-N Emissions

**Figure 5:**
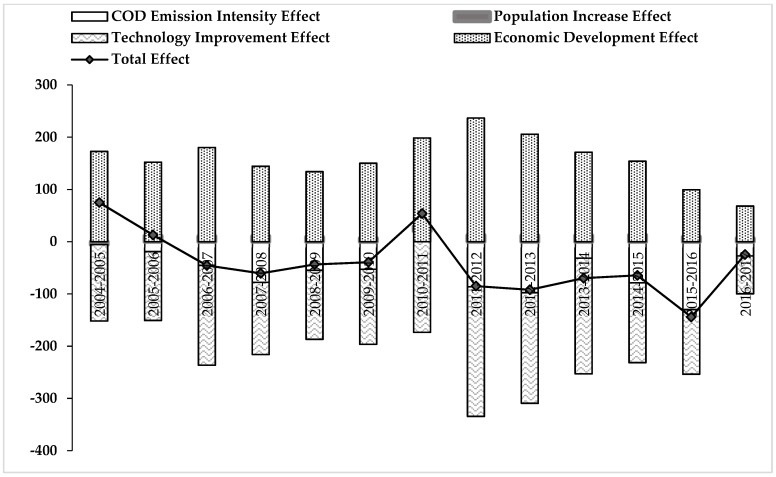
The decomposition analysis results of COD emission effects in mainland China from 2004 to 2017.

**Figure 6:**
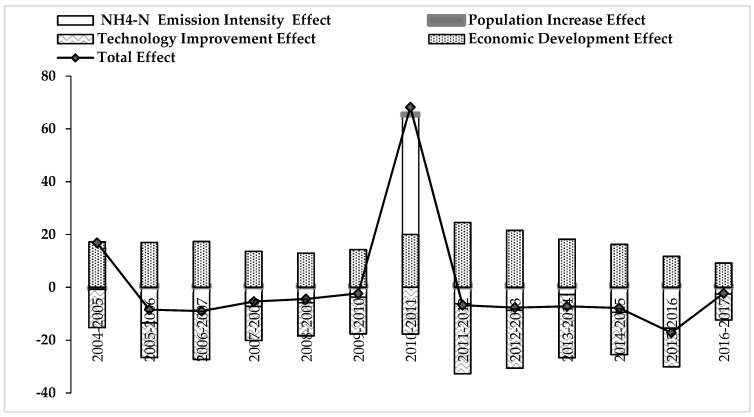
The decomposition analysis results of NH_4_-N emission effects in mainland China from 2004 to 2017.

By conducting a time series of LMDI decomposition analysis, the driving factors for point source pollutants in mainland China have been quantified. Figure 5 and Figure 6 present the decomposition analysis results of point source pollution of COD and NH_4_-N emissions in China from 2004 to 2017, in which it is clear that the total effect of the emissions showed a downwards trend.

According to the results, economic growth is the main driving factor for the growth of COD emissions. The cumulative effect of economic development on pollution increase reaches 2067.61, with a relative contribution rate of 392.31% and an absolute contribution rate of 42.21% from 2004 to 2017 (See Appendix A Table A1). Though the effect of population growth also promotes the rise of COD emission levels to a certain extent, the absolute contribution value is less than 2.41%, indicating that the population factor had a small impact on the growth of COD emission effects. Wastewater treatment technology improvements and COD emission intensity are the main factors that curbed the rise of total pollution effect. This result concurs with the work of Zhang [49]. The effect of technological progress and COD emission intensity reached −2087.22 and −625.68, respectively. Their relative contribution rates are 396.03% and 119.76%, while their absolute contribution rates are 42.61% and 12.77%, respectively. Additional ways for maintaining the declining emission trend of COD levels needs to be given more attention, considering that a water consumption reduction strategy at the cost of economic growth is not in line with the sustainable development principles [50]. Therefore, strategies of improving water use efficiency, and optimizing and upgrading industrial structures need to be designed.

The decomposition analysis results of NH_4_-N emission effects are shown in Figure 6. The trends observed are almost the same as those of the COD emission effects. Economic growth and increases in the population are the main driving factors for NH_4_-N emission effects, while the technology improvement effect and NH_4_-N pollutant emission intensity effect are the primary and secondary factors limiting its rise. The relative contribution rates of the NH_4_-N emission intensity is as small as 0.84%. At the same time, the relative contribution of the impact of economic development (47.83%) and population increases (2.92%) are relatively high, causing the overall total effect’s fluctuation (See Appendix A Table A2). Therefore, how to improve the efficiency of NH_4_-N treatment is challenging.

Further, it is worth noting that the total effect of COD and NH_4_-N emissions showed a decline from 2004 to 2005 and from 2010 to 2011. This is corresponding to the development of infrastructure investment, the Songhua River Pollution incident and China’s multiple investment programs analyzed above. As a result, the positive information is that the total effect of COD and NH_4_-N emissions have continued to fall since 2011, which means that the emission reduction policies have achieved the desired outcomes they previously planned.

The fluctuation of COD and NH_4_-N emission effects is related to policy orientation. In recent years, different levels of government have advocated for sewage emission reductions. They have actively built various sewage treatment plants and even unified sewage treatment plants in some industrial parks to reduce the cost of sewage treatment. All of these policies are conducive to the efficient use of water and sewage treatment effects. However, in order to improve the efficiency of water resource reuse, a large amount of scientific and technological investment is needed, which is time-consuming and laborious for enterprises. Therefore, in order to prevent the above situation, proper tax credits or subsidies need to be given to industrial enterprises with sound pollution abatement schemes and punitive measures taken for industrial enterprises who poorly perform in pollution prevention [51,52].

#### 3.2.2. The Spatial Decomposition Analysis of COD and NH_4_-N Emissions in Different Provinces and Municipalities of China

**Figure 7:**
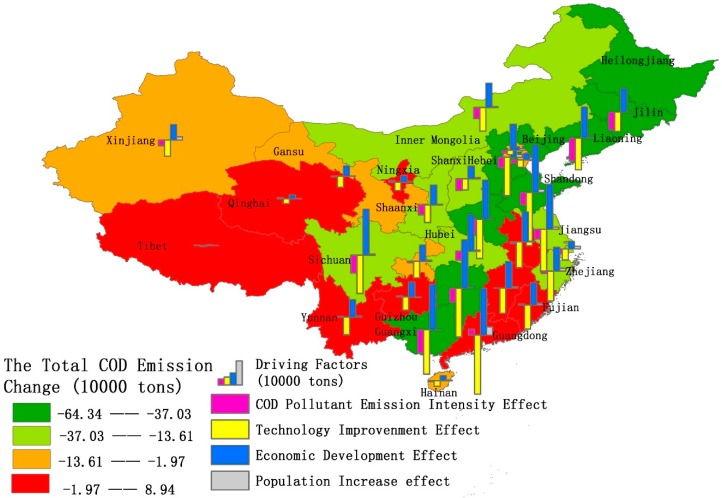
The distribution of COD emissions and contribution of each decomposition driving factor in mainland China from 2004 to 2017.

**Figure 8:**
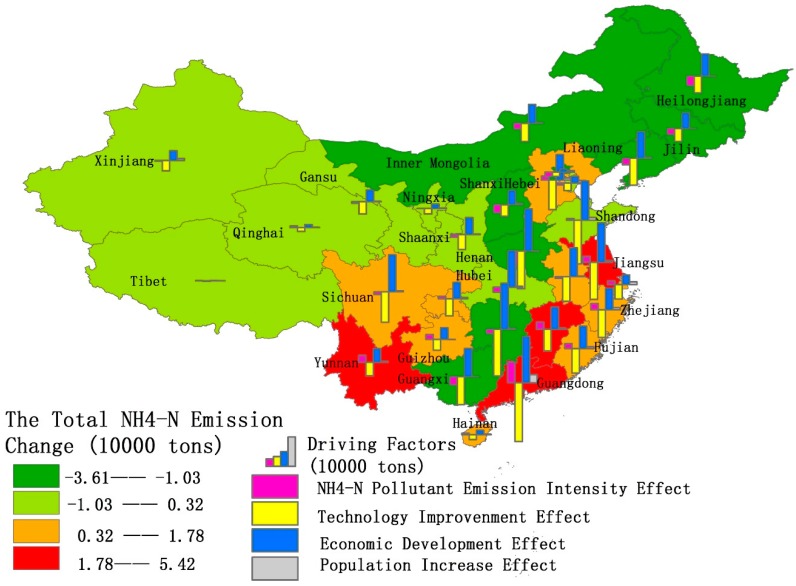
Distribution of NH_4_-N emissions and contributions of each decomposition driving factor in China from 2004 to 2017.

Based on the data of the 31 provinces and municipalities of mainland China from 2004 to 2017, an LMDI decomposition was performed to calculate the spatial effects of COD discharge and NH_4_-N emissions (See Figure 7 and Figure 8, Appendixe A Table A3 and Table A4). The cumulative value of all factors on the level of the COD emission effect is negative for 22 provinces. Only nine provinces and municipalities have a positive value. This means that most provinces and municipalities have achieved progress in the reduction of the COD emission effect. Among these regions, the top three provinces and municipalities with the largest emission effect drop are Guangxi, Liaoning, and Heilongjiang, while the three with the smallest decline in emission effect are Jiangxi, Anhui, and Guizhou. The result might be related to the proportion of secondary industry (which refers to the industry and construction sector) in these areas [53]. In 2017, the proportion of secondary industry in Jiangxi, Anhui, and Guizhou was as high as 48.12%, 47.52% and 41.69%, respectively. This exceeded the national average of 40.66%, while Guangxi, Liaoning, and Heilongjiang, the proportion of the secondary industry was lower. In particular, in Heilongjiang province, secondary industry accounted for only 25.53%.

At the same time, it can also be seen that technology improvements have a greater impact on the cumulative impact in provinces and municipalities, where the proportion of secondary industry is large. Shaanxi, Fujian, Anhui, and Henan are examples of such provinces. Conversely, for provinces and municipalities with a small proportion of the secondary industry, technological improvements have a small impact on total emissions reduction. Beijing and Hainan’s proportion of secondary industry ranks as the last two in mainland China; the role of technological improvement ranks 30th and 28th, respectively.

Figure 8 shows the state of point source NH_4_-N emission effects in mainland China. Sixteen provinces and municipalities have negative values, indicating the net total effect of the positive and negative reinforcing factors have resulted in a decrease in pollutant emissions. The rest of the provinces have positive values, which indicates that NH_4_-N emission curbing efforts need to be strengthened. The top three provinces and municipalities with the largest drop in emissions are Henan, Guangxi, and Liaoning, while the three provinces and municipalities with the smallest emission declines are Guangdong, Jiangsu, and Jiangxi. The result is similar to the total effect of COD emissions as mentioned above. Guangxi, Liaoning, and Jiangxi enjoy nearly the same rank in the total effect of COD discharge and NH_4_-N emissions. Meanwhile, Guangdong ranked fourth from the bottom in net total COD emissions, while Henan ranked fourth. Therefore, Guangxi, Liaoning, Jiangxi, Guangdong and Henan have similar characteristics in COD discharge and NH_4_-N emission effects. Interestingly, the total effect of COD emission in Jiangsu is −15.65, whereas that of NH_4_-N is 3.01. This shows that although Jiangsu has strengthened the treatment of point source pollution and achieved results in COD pollution, the treatment of NH_4_-N emission is still insufficient. NH_4_-N, mainly coming from synthetic fertilizers, will reduce the concentration of dissolved oxygen in the water, leading to a decline in water quality and the death of aquatic plants and animals [54]. Jiangsu, as the province of thousands of lakes and rivers, needs to strengthen NH_4_-N pollution controls to protect the aquatic environment [55].

From the perspective of the four decomposition effects, only eight provinces and municipalities have a positive effect on COD emissions, while as many as 15 provinces and municipalities have registered improvements in NH_4_-N emission effect decreases. This shows that each province pays much more attention to COD emissions compared to NH_4_-N emissions.

The biggest absolute contribution rate area of the COD discharge intensity effect and NH_4_-N emission intensity effect are provinces like Shanxi, Heilongjiang, and Jilin, which have the largest pollution emission intensities. Also, Jiangxi, Fujian, and Yunnan provinces are water scarce areas. This result is strongly related to the degree of water resource endowment. The water resources per capita in Shanxi, Heilongjiang and Jilin rank 5th, 3rd and 13th respectively in the country. Therefore, abundant water resource endowments will be beneficial in containing COD and NH_4_-N emission quantities.

Regarding the role of technology improvements in curbing COD and NH_4_-N emission effects, Zhejiang, Gansu, and Guangdong provinces take the top three ranks. On the contrary, Heilongjiang, Shanxi, and Jilin occupy the bottom three places. This result is in line with the realization that Zhejiang and Guangdong provinces have strengthened water pollution management controls in recent years, not only through the establishment of a stricter water resource management system, such as the River and Lake Chief System, but also through the promotion of water pollution technology to strengthen water treatment and reduce COD and NH_4_-N emissions.

When we look at the impact of economic development, COD and NH_4_-N emission, Anhui, Jiangxi and Guizhou are highly impacted, while Beijing, Shanghai and Tianjin are barely impacted. According to the GDP growth rate in 2017, Guizhou (10.2%), Jiangxi (8.8%) and Anhui (8.46%) ranked among the top five across the 31 provinces and municipalities, while Beijing (6.74%), Tianjin (3.64%) and Shanghai (6.9%) ranked among the bottom eight provinces and municipalities. In particular, Tianjin ranked second from the bottom with a GDP growth rate of only 3.64%. This indicates that the higher the level of economic development, the amount of COD discharge and NH_4_-N emission effects will be higher.

Regarding the net impact of population increases on COD and NH_4_-N emissions, Beijing, Tianjin, and Shanghai occupy the top three and Jilin, Henan and Heilongjiang are positioned in the bottom three. This result is consistent with changes in the net population flow. Compared to 2014, the total population of Beijing, Tianjin and Shanghai increased by 45.39%, 52.05%, and 31.77% respectively in 2017, ranked in the top three in terms of the population growth rate, while Jilin, Henan and Heilongjiang are in the bottom five, especially Henan and Heilongjiang which showed negative population growth. It can be seen from the results that population migration brings little influence on the net COD discharge and NH_4_-N emission effects. Although there is an increase in population density, this makes it convenient for centralized treatment [56,57]. This is the reason why, even though there is a significant increase in population, increases in the emission effects of COD and NH_4_-N are not significant.

#### 3.2.3. The Spatial Decomposition Analysis of COD and NH_4_-N Emissions Effects in Eastern, Central and Western Regions of Mainland China

As can be seen from the decomposition results of the influencing factors of changes in the COD emission effects in the eastern, central and western regions, all these regions have been reducing their COD and NH_4_-N emissions. Among them, the central part has the most significant overall decline in the COD emission effect with a value as high as 33.209 (See Table 2). All the pollutant emission influencing factors considered in this article have the same impacts on COD emission in the central and western regions. The variance of the COD emission intensity effect is the reason for the difference in total effect in the central and western regions. Furthermore, the effect of technology improvements in the three regions is negative, indicating that the efficiency of sewage treatment has generally improved and significant progress has been made in the technology and management of industrial sewage treatment [58]. However, by observing the COD total effect in the three regions, it can be easily seen that the eastern and central regions have greater advantages than the western regions. Meanwhile, because of the surging economic growth in the central region, the COD emission caused by economic development is much higher than that in the eastern and western regions, which shows that balancing the economic development and environmental pollution control variables is still a key issue that needs to be given attention in the central region [59]. The effect of population growth in different regions on COD emissions is positive but not significant. This shows that the net population growth in the eastern region is higher than that in the central and western regions.

Likewise, the NH_4_-N emission effect trend changes in the eastern, central and western regions are shown in Table 3. Apart from the central region which has a declining trend, the total effect of NH_4_-N emissions increased by varying degrees in other regions. The economic development effect, population increase effect, and technology improvement effect have consistent impacts on the NH_4_-N emission effect in central and western China, while the disparity in NH_4_-N emission intensity is the main reason for the difference between the central and western parts of the country.

In the eastern and western regions, in addition to improving sewage treatment technology in order to reduce the NH_4_-N emissions, water saving policies need further improvement. Moreover, the economic development effect, population increase effect and technology improvement have the same influences on NH_4_-N emission decreases. Comparatively speaking, the eastern and central regions have greater advantages than western region in sewage treatment technology and the relatively rapid economic development brings more NH_4_-N emissions to the central region. At the same time, the population increase effect is in line with the actual situation. Population growth in the east is higher than that in the central and western regions. As a result, the total emission effect of NH_4_-N in the east outweighs those in other regions.

## 4. Conclusions and Recommendation

### 4.1. Conclusions

In this paper, the LMDI method was used to decompose the effects of the main determining factors of point source COD and NH_4_-N pollution in mainland China from 2004 to 2017. The four main emission influencing factors are taken into account (pollutant emission intensity effect, technology improvement effect, economic development effect, and population increase effect).

(1) The temporal characteristics of COD and NH_4_-N emissions are similar. Both COD discharges and NH_4_-N emissions showed a steady downtrend from 2004 to 2017. Technology improvement is the main factor that inhibited the increment of COD and NH_4_-N emission effects. Pollutant emission intensity is conducive to the reduction of COD and NH_4_-N emissions. Economic development plays a significant role in the rise of COD and NH_4_-N emissions, while population increases exerts a little effect.

(2) The spatial features of the COD discharge and NH_4_-N emission effects show a regional difference in the provinces and municipalities of mainland China. The reduction of the COD emission effect in each province and municipality is obviously better than that of the NH_4_-N emission effect. Except for nine provinces and municipalities, 22 provinces and municipalities showed a declining trend in the COD emission effect. However, only 15 provinces and municipalities showed a downtrend of the NH_4_-N emission effect; more than half of the total provinces and municipalities’ NH_4_-N emission effects increased during the study period.

In addition, the influencing degree of the COD discharge and NH_4_-N emission factors are also determined in this article. Among all of them, economic development leads to a rise in COD and NH_4_-N emissions in all provinces and municipalities. Population increases had little effect on the increase of COD and NH_4_-N emissions. On the contrary, improvements in water use and treatment technology are the main restricting factors for COD and NH_4_-N emissions. In most provinces and municipalities, this effect has a negative reinforcing impact on the COD emission effect. On the other hand, in nearly half of the provinces and municipalities, this effect has a positive reinforcing influence on the NH_4_-N emission effect.

(3) In the eastern, central and western areas of mainland China, the overall effect of COD emissions showed a downwards trend and the total emissions of NH_4_-N of the central area depicted a decreasing trend as well. However, the eastern and western areas appeared to follow a rising trend. The technology improvement effect, economic development effect and population effect have the same impacts in the eastern, the central and the western areas on COD discharge and NH_4_-N emissions. The variance of the pollutant emission intensity effect is the reason leading to the great differences among the three areas. In terms of sewage technology, the eastern and central regions have more advantages than the western region in sewage treatment technology. The economic growth of the central region brings more COD discharge and NH_4_-N emission effects comparatively. Since the population growth in the eastern region is higher than that in the central and western regions, the COD discharge and NH_4_-N emission effects increased significantly higher compared to the central and western regions.

### 4.2. Recommendations

Based on the results we put forward the following suggestions: firstly, technological progress is the main factor restricting the growth of COD and the NH_4_-N emission effects, so the central and local governments need to increase additional pollution treatment investments. In this way, enterprise-led investment in pollution treatments can be driven by the realization of government-led investment in pollution treatment, so as to promote water utilization and wastewater treatment, which leads to the decrease of COD and NH_4_-N emissions.

Secondly, the pricing reform of water resources needs to be strengthened in regions which are rich in water resources to raise the awareness of water conservation in these regions and improve water use efficiency.

Lastly, in order to fully tap the water-saving potential of the central and western regions, the developed eastern region needs to strengthen its technological, managerial and financial support for the less developed central and western regions.

## Figures and Tables

**Table 1:** Division of provinces and municipalities in mainland China.

Provinces	Municipalities
Hebei, Shanxi, Inner Mongolia, Liaoning, Jilin, Heilongjiang, Jiangsu, Zhejiang, Anhui, Fujian, Jiangxi, Shandong, Henan, Hubei, Hunan, Guangdong, Guangxi, Hainan, Sichuan, Guizhou, Yunnan, Xizang, Shanxi, Gansu, Qinghai, Ningxia, Xinjiang	Beijing Tianjin Shanghai Chongqing

**Table 2:** The decomposition analysis results of COD emission in mainland China of Eastern, Central and Western regions from 2004 to 2017.

Region	Total Effect	COD Emission Intensity Effect	Technology Improvement Effect	Economic Development Effect	Population Increase Effect
Eastern region	−15.794	−15.212	−77.411	69.926	6.904
Central region	−33.209	−40.834	−86.224	92.500	1.350
Western region	−6.531	−10.587	−42.590	44.410	2.236

**Table 3:** The decomposition analysis results of NH_4_-N emission in mainland China of Eastern, Central and Western regions from 2004 to 2017.

Region	Total Effect	NH_4_-N Emission Intensity Effect	Technology Improvement Effect	Economic Development Effect	Population Increase Effect
Eastern Region	0.847	0.936	−8.424	7.564	0.771
Central Region	−1.078	−1.999	−8.693	9.434	0.180
Western Region	0.457	0.080	−4.157	4.319	0.215

## Abbreviations

COD	Chemical oxygen demand;
NH_4_-N	Ammonia nitrogen;
LMID	Logarithmic Mean Divisia Index.

## Nomenclature

**Symbol**	**Definition**
i	It refers to the corresponding provinces or municipalities
n	The total sum of provinces and municipalities
t	It refers to the target year.
m	It refers to the base year.
$W^{t}$	It represents the total number amount of pollutant (COD or NH_4_-H) that emitted in year throughout the country.
$V_{i}$	It represents the water resource consumption in the province i
$G_{i}$	It represents the gross regional product
$P_{i}^{t}$	It represents the total population in the province i
$W_{S,i}$	It represents the pollutant emission intensity
$W_{T,i}$	It depicts the wastewater technology improvement effect
$W_{E,i}$	It denotes the impact of economic development
$W_{P,i}$	It represents the population increase effect
$\omega_{S},\omega_{T},\omega_{J},\omega_{P}$	It represents the relative contribution rate of each factor
$\Delta W_{R,i}$	It reflects the relative total sum of each factor’s effect.
$\lambda_{S},\lambda_{T},\lambda_{J},\lambda_{P}$	It represents the absolute contribution rate of each factor
$\Delta W_{A,i}$	It reflects the absolute total sum of each factor’s effect.

## Appendix A

**Table A1:** The decomposition analysis results of COD emission in mainland China from 2004 to 2017.

Time Series	Total Effect	COD Emission Intensity Effect	Technology Improvement Effect	Economic Development Effect	Population Increase Effect
2004–2005	54.05	−146.38	172.82	−5.46	75.02
2005–2006	−18.83	−131.86	152.28	11.30	12.90
2006–2007	−45.22	−191.22	180.25	10.69	−45.50
2007–2008	−77.82	−138.06	144.27	11.01	−60.60
2008–2009	−54.63	−132.15	134.06	9.23	−43.50
2009–2010	−52.73	−143.78	150.39	6.82	−39.30
2010–2011	20.50	−173.50	198.23	8.57	53.79
2011–2012	−86.22	−248.07	236.64	12.67	−84.98
2012–2013	−97.64	−211.64	205.48	11.86	−91.94
2013–2014	−31.40	−221.57	171.41	11.82	−69.73
2014–2015	−78.55	−152.96	154.07	13.01	−64.43
2015–2016	−129.92	−123.62	99.51	9.82	−144.21
2016–2017	−27.27	−72.40	68.21	6.90	−24.56
Sum effect	−625.68	−2087.22	2067.61	118.24	−527.04
The relative contribution rate	---	118.72%	396.03%	392.31%	22.44%
The absolute contribution rate	1	12.77%	42.61%	42.21%	2.41%

**Table A2:** The decomposition analysis results of NH_4_-N emission in mainland China from 2004 to 2017.

Time Series	Total Effect	NH_4_-N Emission Intensity Effect	Technology Improvement Effect	Economic Development Effect	Population Increase Effect
2004–2005	14.92	−14.55	17.17	−0.69	16.86
2005–2006	−13.42	−13.14	16.96	1.14	−8.46
2006–2007	−8.69	−18.62	17.34	0.99	−8.98
2007–2008	−7.16	−12.94	13.60	1.15	−5.36
2008–2009	−5.80	−12.57	12.95	1.03	−4.39
2009–2010	−3.67	−13.93	14.26	1.04	−2.30
2010–2011	65.00	−17.68	19.99	0.92	68.22
2011–2012	−6.21	−26.49	24.55	1.40	−6.75
2012–2013	−8.64	−21.92	21.58	1.30	−7.67
2013–2014	−2.70	−23.93	18.18	1.28	−7.18
2014–2015	−9.39	−16.10	16.24	1.42	−7.82
2015–2016	−15.43	−14.65	11.72	1.18	−17.18
2016–2017	−2.53	−9.85	9.20	0.91	−2.27
Sum effect	−3.73	−216.35	213.74	13.06	6.71
The relative contribution rate	---	55.61%	3223.26%	3184.36%	194.51%
The absolute contribution rate	1	0.84%	48.41%	47.83%	2.92%

**Table A3:** The decomposition analysis results of COD emission in mainland China from 2004 to 2017.

Provinces and Municipalities	Total Effect	COD Emission Intensity Effect	Technology Improvement Effect	Economic Development Effect	Population Increase Effect
Beijing	−6.05	−7.91	−12.54	9.82	4.59
Tianjin	−6.46	−9.89	−20.64	17.57	6.51
Hebei	−37.43	−29.94	−107.12	91.66	7.98
Shanxi	−23.64	−32.15	−30.77	35.68	3.59
Inner Mongolia	−27.30	−31.08	−65.95	67.37	2.36
Liaoning	−63.68	−63.76	−88.58	86.38	2.27
Jinlin	−38.09	−50.33	−54.28	66.20	0.30
Heilongjiang	−55.80	−74.92	−71.39	91.42	−0.90
Shanghai	−16.35	−13.72	−30.78	20.66	7.48
Jiangsu	−15.65	−27.27	−119.30	124.89	6.02
Zhejiang	−16.02	−6.84	−83.07	66.12	7.77
Anhui	8.67	−4.88	−71.61	83.28	1.88
Fujian	2.96	2.79	−66.62	61.84	4.95
Jiangxi	8.94	0.43	−69.85	74.20	4.16
Shandong	−41.85	−35.12	−148.52	132.80	8.99
Henan	−42.09	−41.44	−109.13	109.18	−0.70
Hubei	−13.61	−24.36	−94.85	102.61	2.99
Hunan	−37.03	−38.68	−135.72	134.42	2.94
Guangdong	4.86	16.54	−164.53	129.95	22.91
Guangxi	−64.34	−65.24	−123.81	126.28	−1.58
Hainan	−2.52	−2.55	−15.63	14.15	1.51
Chongqing	−1.97	−4.07	−46.27	45.34	3.02
Sichuan	−24.97	−49.83	−106.42	128.26	3.03
Guizhou	5.15	2.84	−37.12	41.12	−1.68
Yunnan	4.73	2.02	−49.03	48.55	3.19
Xizang	1.13	0.90	−2.07	1.95	0.36
Shanxi	−19.54	−27.46	−48.97	55.35	1.54
Gansu	−3.30	−1.77	−30.90	28.52	0.85
Qinghai	1.67	3.26	−13.12	10.64	0.88
Ningxia	2.31	4.67	−23.34	18.60	2.37
Xinjiang	−9.77	−15.94	−45.30	42.81	8.66
Gross effect	−527.04	−625.68	−2087.22	2067.61	118.24

**Table A4:** The decomposition analysis results of NH_4_-N emission in mainland China from 2004 to 2017.

Provinces and Municipalities	Total Effect	NH_4_-N Emission Intensity Effect	Technology Improvement Effect	Economic Development Effect	Population Increase Effect
Beijing	−6.05	−7.91	−12.54	9.82	4.59
Tianjin	−6.46	−9.89	−20.64	17.57	6.51
Hebei	−37.43	−29.94	−107.12	91.66	7.98
Shanxi	−23.64	−32.15	−30.77	35.68	3.59
Inner Mongolia	−27.30	−31.08	−65.95	67.37	2.36
Liaoning	−63.68	−63.76	−88.58	86.38	2.27
Jinlin	−38.09	−50.33	−54.28	66.20	0.30
Heilongjiang	−55.80	−74.92	−71.39	91.42	−0.90
Shanghai	−16.35	−13.72	−30.78	20.66	7.48
Jiangsu	−15.65	−27.27	−119.30	124.89	6.02
Zhejiang	−16.02	−6.84	−83.07	66.12	7.77
Anhui	8.67	−4.88	−71.61	83.28	1.88
Fujian	2.96	2.79	−66.62	61.84	4.95
Jiangxi	8.94	0.43	−69.85	74.20	4.16
Shandong	−41.85	−35.12	−148.52	132.80	8.99
Henan	−42.09	−41.44	−109.13	109.18	−0.70
Hubei	−13.61	−24.36	−94.85	102.61	2.99
Hunan	−37.03	−38.68	−135.72	134.42	2.94
Guangdong	4.86	16.54	−164.53	129.95	22.91
Guangxi	−64.34	−65.24	−123.81	126.28	−1.58
Hainan	−2.52	−2.55	−15.63	14.15	1.51
Chongqing	−1.97	−4.07	−46.27	45.34	3.02
Sichuan	−24.97	−49.83	−106.42	128.26	3.03
Guizhou	5.15	2.84	−37.12	41.12	−1.68
Yunnan	4.73	2.02	−49.03	48.55	3.19
Xizang	1.13	0.90	−2.07	1.95	0.36
Shanxi	−19.54	−27.46	−48.97	55.35	1.54
Gansu	−3.30	−1.77	−30.90	28.52	0.85
Qinghai	1.67	3.26	−13.12	10.64	0.88
Ningxia	2.31	4.67	−23.34	18.60	2.37
Xinjiang	−9.77	−15.94	−45.30	42.81	8.66
Gross effect	−527.04	−625.68	−2087.22	2067.61	118.24
